# Enhanced control of continuous stirred tank reactor with two-degree-of-freedom PID driven by Kirchhoff’s law algorithm

**DOI:** 10.1038/s41598-026-44778-w

**Published:** 2026-03-27

**Authors:** Gökhan Yüksek, Serdar Ekinci, Musa Yılmaz

**Affiliations:** 1 Department of Electrical and Electronics Engineering, Batman University, Batman, 72100 Turkey; 2 Department of Computer Engineering, Bitlis Eren University, 13100 Bitlis, Turkey; 3 Center for Environmental Research and Technology, Bourns College of Engineering, University of California at Riverside, Riverside, CA 92521 USA

**Keywords:** Kirchhoff’s law algorithm, Continuous stirred tank reactor, Artificial intelligence, 2DOF-PID Control, Energy science and technology, Engineering, Mathematics and computing, Physics

## Abstract

This study examines the use of Kirchhoff’s law algorithm (KLA) to calibrate a two-degree-of-freedom PID (2DOF-PID) controller for temperature regulation in a non-linear continuous stirred tank reactor (CSTR). The reactor model shows strong thermal nonlinearity. It also shows a noticeable time delay. This is based on the coupled mass and energy balance equations. This makes accurate temperature control much more difficult. These effects were addressed by KLA, which is formulated as a physics-based optimization method rather than a learning or data-driven method. The algorithm produced a set of controller gains that yielded faster, more stable responses by treating the current–voltage equilibrium of electrical circuits as an energy-balanced search method. The optimally tuned 2DOF-PID controller has lower rise and settling times, and minimal steady-state error, compared with the other standard optimizers tested in an identical manner. The results show that combining KLA with controller design can improve both robustness and control accuracy for non-linear thermal systems.

## Introduction

Temperature control is among the key challenges in chemical reactor operation, as it directly influences energy conversion efficiency and product quality^1,2^. Continuous stirred tank reactors (CSTRs) are difficult to control due to high heat transfer rates, time-varying reaction kinetics, and strong nonlinearities. Small temperature changes can affect reaction rates and equilibrium states, resulting in changes in product selectivity and instability of the entire process^3,4^. It is thus necessary to keep the reactor temperature within a narrow range to guarantee process safety and energy efficiency^5^. However, the intrinsic time delays and parameter variations in the system are generally much larger than what traditional control methods can handle^6^.

Proportional–integral–derivative (PID) controllers are still the most widely used for CSTR temperature control^6–12^. However, their tuning is usually derived from linear models, which restrict their performance in non-linear operating conditions. The proportional, integral, and derivative terms are all coupled, which can lead to the control behaving very differently at various steady states. Furthermore, employing the same control route for both reference tracking and disturbance rejection introduces a trade-off: the controller is either fast with overshoot or slow but conservative. This natural trade-off limits the performance of single-degree designs for CSTR systems with multi-stage thermal dynamics^6^.

Two degrees of freedom PID controllers (2DOF-PID) were developed to address the limitations of classic PID configurations by separating tracking and regulation loops while preserving a familiar formulation^13^. Thanks to the two new coefficients $\beta$ and $\gamma$, the response to reference changes can now be tuned independently, potentially allowing faster rise times and smaller overshoots^14,15^. However, with these additional parameters, the search space of controller gains is enlarged and manual tuning becomes impractical, thus optimization-based design becomes necessary. In time-delay or multivariable systems, it is hard to select all the gains in a balanced manner using deterministic tuning rules^16^. Since it retains the simplicity and industrial applicability of classical PID controllers while introducing more degrees of freedom by independently tuning the tracking and regulating paths, the 2DOF-PID structure was preferred in this study. Although advanced techniques such as model predictive control or fractional-order PID controllers can provide even more flexibility, they usually require greater computational effort or introduce additional parameters that complicate implementation.

Recently, natural- or heuristic-process-based optimization algorithms have gained greater acceptance in control applications due to their ability to search effectively in non-linear solution spaces. These approaches typically show good convergence but may suffer from premature convergence or an unbalanced trade-off between exploration and exploitation. When used across various control tasks, their robustness is often challenged by their strong dependence on algorithm-specific parameters. Therefore, there is still a need for a more stable, well-balanced, and parameter-free search method capable of handling multi-parameter controllers, such as the 2DOF-PID^17^.

In accordance with this stipulation, a control configuration founded on Kirchhoff’s law algorithm (KLA) has been adopted to enhance the gain parameters of a 2DOF-PID controller. In accordance with the tenets of current and voltage balance that underpin the analysis of electrical circuits, KLA employs a modelling approach to depict the energy interaction between solutions in the optimisation process. This is achieved by simulating the equilibrium conditions of circuit currents^18^. Information transfer between different regions of the population is possible via this physics-based approach, which facilitates energy flow. This enables a natural balance between the global search and local refinement stages. The balancing mechanism created by this, under the complex thermal response of the CSTR system, makes the parameter space stable and supports low-oscillation convergence behaviour.

Recent studies on the temperature control of CSTR have focused mainly on improving the performance of PID-based and hybrid controllers through evolutionary and model-based optimization frameworks. Deifalla and Abdalla developed a Genetic Algorithm tuned PID control system for an exothermic CSTR process, achieving substantial reductions in rise time, overshoot, and settling time compared to classical Ziegler–Nichols and Tyreus–Luyben tuning methods. However, their approach remained confined to idealized simulations and a single reaction system^19^. In a more advanced study, Wang et al. integrated an improved firefly algorithm for parameter identification with a hybrid sparrow search algorithm–PID framework, yielding superior robustness and dynamic accuracy under nonlinear and time-delayed conditions^20^. Meanwhile, Kumavat and Thale examined hybrid MPC–PID configurations for CSTR temperature regulation, demonstrating smoother responses and lower overshoot than standalone PID or MPC schemes, highlighting the value of model-predictive integration^21^. Govind et al. proposed a nonlinear constraint–based decentralized PID framework for benchmark CSTR systems, where the controller gains were optimized through frequency-domain constraints on amplitude ratio and bandwidth while ensuring robust stability using the Kharitonov–Hurwitz theorem^22^. This approach effectively reduced loop interactions and achieved fast servo and regulatory responses across multiple operating regions. However, the design remained highly model-dependent and required manual specification of stability margins. On the other hand, Ajlouni et al. propose a hybrid PID-tuning scheme that fuses GA/PSO with a machine-learning surrogate so the optimizer can predict fitness and adapt gains on-the-fly, which accelerates convergence and strengthens disturbance-robustness; they further cast tuning as a min–max problem against worst-case plant scenarios to harden the controller under uncertainty^23^.

However, these methods generally depend on either parameter-specific tuning or heuristic mechanisms that require empirical adjustment. None explicitly exploits the physical principles of energy balance and current flow to guide the optimization process. In contrast, the present study uses the KLA which is a parameter-free, physically grounded optimizer that interprets solution interactions through current–voltage equilibrium analogies. By coupling KLA with a 2DOF-PID structure, a self-balanced and low-oscillation control behavior is achieved for the nonlinear thermal dynamics of the CSTR.

The purpose of this study is to establish a fast and smooth control strategy that can precisely follow the temperature variations of a CSTR system. For this purpose, the performance of the KLA-optimized 2DOF-PID controller was compared with the animated oat optimization algorithm (AOO)^24^, parrot optimizer (PO)^25^, coati optimization algorithm (COA)^26^, and dwarf mongoose optimization (DMO)^27^ algorithms. Each method was looked at using the same number of people and repeats, and the checks were done 25 times. The KLA has been found to produce lower objective function values, whilst also enabling the rapid and uniform regulation of the reactor’s temperature. The results, taken as a whole, demonstrate that utilising KLA in conjunction with the 2DOF-PID framework is an effective method of controlling nonlinear thermal processes without the need for any adjustments to be made to the settings.

The main contributions of this study can be summarized as follows:

1. A physics-consistent equilibrium-driven optimization framework is integrated into non-linear thermal control design, introducing a circuit-law-based interpretation of controller-gain interactions.
2. The study demonstrates how Kirchhoff’s current balance principle can be translated into a structured search mechanism that enforces energy-consistent gain adaptation in a strongly non-linear CSTR model.
3. Unlike conventional metaheuristic tuning approaches, the proposed framework eliminates algorithm-specific hyperparameters while maintaining convergence stability, thereby improving repeatability and robustness of controller design.
4. A comprehensive statistical and time-domain evaluation under identical experimental conditions establishes not only performance superiority but also reduced dispersion and improved consistency across independent runs.

## Modeling of a continuous stirred tank reactor

**Fig. 1:**
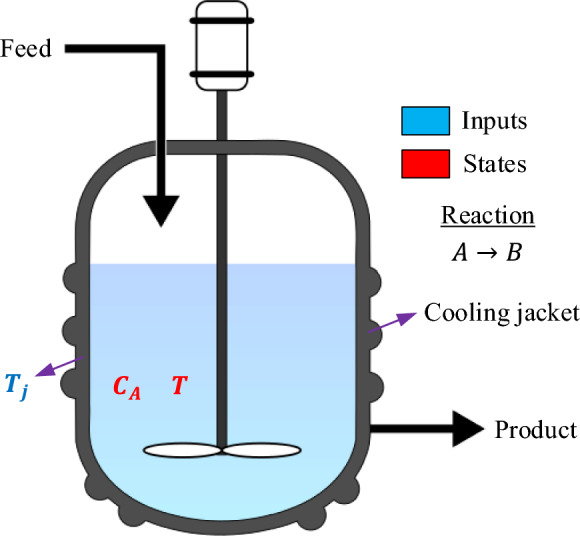
Continuous stirred tank reactor with cooling jacket.

The CSTR is one of the most widely used reactor setups in industrial chemical procedures. As demonstrated in Fig. 1, the configuration comprises a stirred reactor vessel fitted with a cooling jacket, a constant inlet flow, and an outlet flow line. Within the reactor, a single exothermic, irreversible first-order reaction occurs. The heat generated by this reaction is continuously removed through a coolant circulating inside the jacket to keep the reactor temperature close to its nominal operating point. When the balance between heat generation and heat removal is disturbed, the reactor may exhibit more than one steady-state condition. This phenomenon reflects the nonlinear and potentially unstable dynamic nature of CSTR systems^28–30^.

The CSTR is modeled as a continuously flowing system in which reactant feed and product discharge coincide. To make the problem analytically tractable, several standard assumptions are adopted. The reactor contents are assumed to be perfectly mixed, implying that the outlet stream has the same temperature and composition as the bulk fluid within the reactor. The operation is carried out at constant volume $V$, and the thermophysical properties, density $\rho$ and specific heat capacity ${c}_{p}$, are considered constant, independent of temperature or composition changes. The jacket temperature ${T}_{j}$ acts as a directly controllable input variable; therefore, a separate energy balance for the jacket is not required. Under these assumptions, the component balance for species can be expressed as Eq. 1

1 $$V\frac{d{C}_{A}}{dt}=F({C}_{Af}-{C}_{A})-rV$$

where ${C}_{A}$ is the concentration of reactant $A$ within the reactor, ${C}_{Af}$ is its concentration in the feed, $F$ is the volumetric flow rate, and $r$ denotes the reaction rate per unit volume. For a first-order irreversible reaction, the reaction rate follows the Arrhenius dependence as Eq. 2.

2 $$r={k}_{0}\mathrm{exp}\left(-\frac{E}{RT}\right){C}_{A}$$

where ${k}_{0}$ is the pre-exponential factor, $E$ is the activation energy, $R$ is the universal gas constant, and $T$ is the reactor temperature. Substituting Eq. (2) into Eq. (1) gives the non-linear concentration dynamics as a coupled function of ${C}_{A}$ and $T$. The thermal dynamics are derived from the total energy balance, which accounts for convective energy transport through the inlet and outlet streams, heat generation from the exothermic reaction, and heat exchange through the jacket wall. Assuming constant $\rho$ and ${c}_{p}$, the energy balance becomes

3 $$V\rho {c}_{p}\frac{dT}{dt}=F\rho {c}_{p}({T}_{f}-T)+(-\Delta H)Vr-UA(T-{T}_{j})$$

where $U$ is the overall heat-transfer coefficient, $A$ is the heat-transfer area, ${T}_{f}$ is the feed temperature, and $\Delta H$ is the heat of reaction. In the considered control configuration, the jacket temperature ${T}_{j}$ represents the manipulated variable used to regulate the reactor temperature. Substituting Eq. (2) for $r$ yields

4 $$V\rho {c}_{p}\frac{dT}{dt}=F\rho {c}_{p}({T}_{f}-T)+(-\Delta H)V{k}_{0}\mathrm{exp}\left(-\frac{E}{RT}\right){C}_{A}-UA(T-{T}_{j})$$

Eq. (1)–(4) collectively describe the non-linear dynamic behavior of the CSTR. The exponential temperature dependence of the reaction rate introduces strong coupling between concentration and temperature, leading to multiple steady states and the potential for thermal runaway in highly exothermic systems. At steady state, the accumulation terms vanish ($d{C}_{A}/dt=0$, $dT/dt=0$), giving

5 $$0=\frac{F}{V}({C}_{Af}-{C}_{A})-{k}_{0}\mathrm{exp}\left(-\frac{E}{RT}\right){C}_{A}$$

6 $$0=\frac{F}{V}({T}_{f}-T)+\frac{-\Delta H}{\rho {c}_{p}}{k}_{0}\mathrm{exp}\left(-\frac{E}{RT}\right){C}_{A}-\frac{UA}{V\rho {c}_{p}}(T-{T}_{j})$$

The simultaneous solution of Eqs. (5) and (6) determine the steady-state values of ${C}_{A}$ and $T$ for given process parameters. Due to the strong nonlinearity of the exponential term, analytical solutions are rarely feasible. A representative set of model parameters used in this study, obtained from the^28^ is given in Table 1.

**Table 1:** Nominal parameters for the CSTR model^28^.

Variable	Value	Variable	Value
Feed flow rate ($F$)	100 L/min	Activation energy over gas constant ($E/R$)	8750 K
Reactor volume ($V$)	100 L	Heat capacity (${c}_{p}$)	0.239 J/g∙K
Feed temperature (${T}_{f}$)	350 K	Density ($\rho$)	1000 g/L
Pre-exponential factor (${k}_{0}$)	7.2 × 10^10^ min^−1^	Overall heat-transfer coefficient × area ($UA$)	50,000 J/min·K
Heat of reaction ($-\Delta H$)	50,000 J/mol	Feed concentration (${C}_{Af}$)	1 mol/L

The steady-state operation points of the CSTR system:T_j_=280 K, T=304.167553089807 K and C_A_ = 0.977403565332 mol/L.

It is worth noting that the non-linear CSTR model employed in this study represents a classical benchmark problem in process control. The steady-state operating conditions and the corresponding linearized state-space representation of this system have been extensively analyzed in the literature. A detailed derivation of the steady-state solution and local stability characteristics of the CSTR model can be found in Bequette^29^. Therefore, the focus of the present work is on optimizing the controller parameters and evaluating the resulting closed-loop performance, rather than repeating the well-established stability derivations of the process model. 

## Kirchhoff’s law algorithm (KLA)

The KLA is a new physics-based optimization approach used in electrical circuit analysis, based on Kirchhoff’s current law (KCL)^18^. The fundamental idea behind KLA is to adapt the principle that the algebraic sum of currents entering and leaving a node in an electrical circuit is zero to an optimization problem. With this approach, each solution in the system is treated as a node voltage; thus, the interactions between solutions are modeled by analogy to the equilibrium conditions of circuit currents.

### Basic principle and equivalent circuit analogy

Low-cost solutions are characterised by low resistances, which leads to increased current flow. The algorithm naturally guides the search towards these favourable regions by following the least-resistance paths. This is analogous to the physical tendency of an electric current to minimise energy dissipation.

In contrast to most metaheuristics, KLA has no external control parameters. Besides the initial population size ${N}_{pop}$, the search is fully self-regulated by the natural relations between current, voltage, and resistance. This makes KLA straightforward, adaptive, and very robust for different kinds of optimization tasks.

### Electrical and mathematical modeling

The optimization dynamics of KLA can be represented through an equivalent resistive circuit composed of voltage sources and resistors. Each node corresponds to a potential solution, while the links between nodes are cost-dependent and modeled as resistors. According to Ohm’s law, the voltage drop across a resistor is

7 $${V}_{R}={I}_{R}\times R$$

where ${V}_{R}$ and ${I}_{R}$ denote voltage and current through resistance $R$, respectively. In the KLA analogy, when a neighboring node exhibits a lower cost value, its corresponding ${R}_{ij}$ decreases, thus increasing the current ${I}_{ij}$ flowing toward it. Since KCL requires that the total current at a node equals zero, a node with four incident branches satisfies

8 $${I}_{1}+{I}_{2}+{I}_{3}-{I}_{4}=0$$

and for the updated system:

9 $${I}_{1}^{new}={I}_{2}^{new}+{I}_{3}^{new}+{I}_{4}^{new}$$

Each branch current is then expressed in terms of nodal voltages:

10 $${I}_{1}^{new}=\frac{{V}_{1}^{new}-{V}_{2}^{new}}{{R}_{12}}+\frac{{V}_{1}^{new}-{V}_{3}^{new}}{{R}_{13}}+\frac{{V}_{1}^{new}-{V}_{4}^{new}}{{R}_{14}}$$

This formulation establishes the physical foundation of KLA’s update equations, translating Kirchhoff’s current balance into a search-space equilibrium process. The resistance between nodes $i$ and $j$ is defined as a random function of their objective values:

11 $${R}_{ij}^{\hspace{0.17em}k}=\frac{1}{(\mathrm{rand}+\mathrm{rand})}{\left(\frac{f({X}_{i}^{k})}{f({X}_{j}^{k})}\right)}^{2\times \mathrm{rand}}$$

where $\mathrm{rand}\sim U(\mathrm{0,1})$ is a uniformly distributed random number. This ensures that links between superior solutions exhibit lower resistance, enabling the current to follow more efficient routes. According to KCL, the net current at node $i$ must vanish, which is reflected in the position update term:

12 $$\Delta {X}_{i}^{k}=\sum_{j=a,b,c}\frac{({X}_{i}^{k}-{X}_{j}^{k})}{{R}_{ij}^{\hspace{0.17em}k}}$$

Substituting Eq. (12) gives the expanded form:

13 $$\begin{array}{cc}\Delta {X}_{i}^{k}=& (\mathrm{rand}+\mathrm{rand}){\left(\frac{f({X}_{a}^{k})}{f({X}_{i}^{k})}\right)}^{2\times \mathrm{rand}}\cdot \frac{f({X}_{a}^{k})-f({X}_{i}^{k})}{\mid f({X}_{a}^{k})-f({X}_{i}^{k})\mid +\varepsilon }({X}_{i}^{k}-{X}_{a}^{k})\\ & +(\mathrm{rand}+\mathrm{rand}){\left(\frac{f({X}_{b}^{k})}{f({X}_{i}^{k})}\right)}^{2\times \mathrm{rand}}\cdot \frac{f({X}_{b}^{k})-f({X}_{i}^{k})}{\mid f({X}_{b}^{k})-f({X}_{i}^{k})\mid +\varepsilon }({X}_{i}^{k}-{X}_{b}^{k})\\ & +(\mathrm{rand}+\mathrm{rand}){\left(\frac{f({X}_{c}^{k})}{f({X}_{i}^{k})}\right)}^{2\times \mathrm{rand}}\cdot \frac{f({X}_{c}^{k})-f({X}_{i}^{k})}{\mid f({X}_{c}^{k})-f({X}_{i}^{k})\mid +\varepsilon }({X}_{i}^{k}-{X}_{c}^{k})\end{array}$$

The coefficient controlling current direction is defined as

14 $$\frac{f({X}_{j}^{k})-f({X}_{i}^{k})}{\mid f({X}_{j}^{k})-f({X}_{i}^{k})\mid +\varepsilon }=\left\{\begin{array}{c}1, f({X}_{i}^{k})<f({X}_{j}^{k})\\ -1, f({X}_{i}^{k})>f({X}_{j}^{k})\\ 0, f({X}_{i}^{k})=f({X}_{j}^{k})\end{array}\right.$$

indicating the current flow from higher-cost to lower-cost nodes. Thus, nodes with lower objective values are high-potential, attracting the search toward global optima.

### Update mechanism and selection strategy

The new position of each node is computed as

15 $${X}_{i,new}^{k}={X}_{i}^{k}+\Delta {X}_{i}^{k}$$

If $f({X}_{i,new}^{k})\le f({X}_{i}^{k})$, the update is accepted; otherwise, the previous position is retained.

After updating all individuals, both the new and old populations are merged, sorted, and the best ${N}_{pop}$ candidates are forwarded to the next iteration.

This formulation can also be interpreted through the energy dissipation law $P={I}^{2}R$ minimizing power loss corresponds to minimizing the objective function, leading the system toward the optimal equilibrium configuration.

### Flow diagram and optimization process

**Fig. 2:**
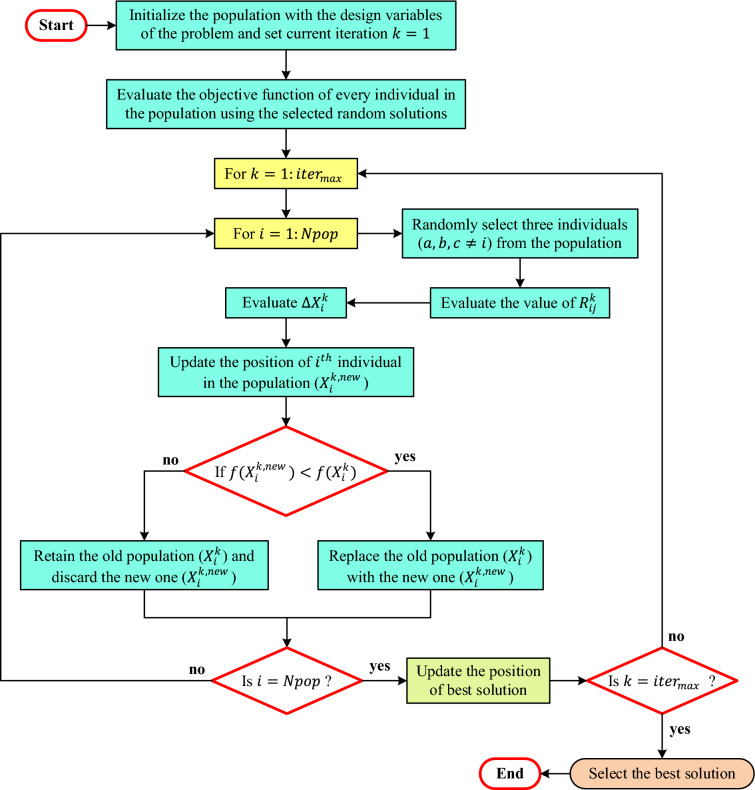
Flowchart of KLA.

The flowchart of the KLA is illustrated in Fig. 2. The algorithm starts with an initial set of ${N}_{pop}$ solution vectors, which in this case are nodal voltages. During each iteration:

1. Three nodes are randomly selected from the sample, and their positions are updated according to the KCL-based model.
2. After evaluating the new solutions, the ones that result in the least power loss are retained.
3. The new generation is combined with the old, and the best ${N}_{pop}$ individuals are selected and propagated to the next generation.

This series of events continues until the maximum number of iterations is reached. In each iteration, the algorithm adjusts the resistances to minimize the overall energy loss, leading the search process toward the minimum of the objective function.

### Parameter-free structure and exploration–exploitation balance

An important distinction of the KLA is that it does not require fixing any rules or strategies in advance. The execution remains simple and uniform across different problems, since the only tuning required is selecting the population size. Due to its operating principle, the algorithm automatically maintains a balance between exploration and exploitation without requiring additional weighting coefficients. While looking, random numbers are used to decide how hard it is to move from one place to another. This helps the group move towards potential good places. The improvement basically works like a current of electricity, trying to reduce energy loss until things are in balance.

### Application of KLA to the optimization problem

KLA is a versatile tool that can be used to solve multidimensional continuous optimization problems in various ways. Each solution vector is interpreted as a nodal voltage. At the same time, the resistance-current relationship is compared to the slope of the cost function. The shift in the algorithm towards the ideal solution is an inevitable outcome of the current trend towards high-cost areas. In this study, the KLA framework is used to determine the optimal 2DOF-PID parameters for the CSTR system. The rules of KCL ensure that the PID gains converge to an energy balance similar to that observed in real systems.

KLA is unique because it uses a physics-based approach, unlike conventional evolutionary or swarm intelligence-based algorithms. KLA only pursues equilibrium through physical laws, so it doesn’t need empirical coefficients. This functionality enables the provision of a highly effective optimisation framework with minimal parameter requirements, exceptional reliability, and extensive compatibility.

## Proposed control design method

The control strategy developed in this study relies on a versatile framework that can adapt to the variable behaviour of non-linear, time-delayed processes. Its main aim is to improve how well the system performs when in use, without making it less stable. A 2DOF-PID configuration is employed for this purpose, with independent tuning of reference tracking and disturbance rejection. Within this structure, the controller’s benefits are determined automatically through optimisation, enabling precise operation even in the presence of delays. The following sections describe the structural layout and mathematical formulation of the 2DOF-PID controller.

### 2DOF-PID controller structure

**Fig. 3:**
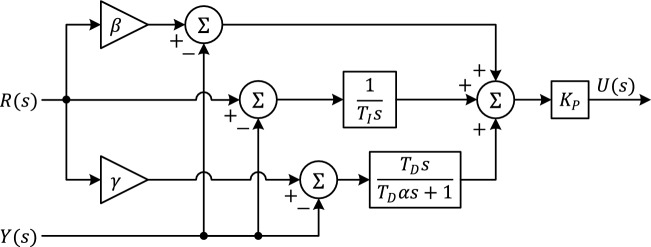
Block diagram of 2DOF-PID controller.

The two-degree-of-freedom PID controller shown in Fig. 3 allows adjusting the aggressiveness of the reference transitions by scaling the tracking and regulation paths with separate gains. The controller output $U(s)$ for the process output $Y(s)$ and the reference $R(s)$ are defined by the following equation:

16 $$U\left(s\right)={K}_{P}\left(\beta R\left(s\right)-Y(s)+\frac{1}{{T}_{I}s}\left[R\left(s\right)-Y(s)\right]+\frac{{T}_{D}s}{{T}_{D}\alpha s+1}\left[\gamma R\left(s\right)-Y(s)\right]\right)$$

Here, ${K}_{P}$ proportional gain, ${T}_{I}$ is the integral time constant, ${T}_{D}$ is the derivative time constant, $\alpha \in \left(\mathrm{0,1}\right]$ is the pole ratio of the derivative filter, $\beta >0$ is the proportional scale in the tracking path, $\gamma >0$ is the derivative scale in the tracking path.

### Scenario and normalization

In the CSTR case, the reactor temperature $T$ was chosen as the controlled variable. Although concentration dynamics are implicitly accounted for in the mass and energy balance equations, temperature is selected as the main controlled variable because it directly influences reaction rate, conversion, and thermal safety in exothermic CSTR operation. A step change in the reference signal from 304.16755 K to 324.16755 K was made at $t = 1$ min, and all simulations were performed for 20 min. To make the comparison among controllers fair, the output responses are normalized with respect to their initial and final steady-state levels, as in Eq. 17.

17 $${y}_{norm}(t)=\frac{y(t)-{y}_{initial}}{{y}_{final}-{y}_{initial}}$$

This normalization allows all temperature trajectories to be expressed in a unit scale (0–1), making it possible to compare the transient dynamics independent of absolute temperature values.

### Performance metrics

Four normalized metrics were used to assess the transient and steady-state behavior of each controller quantitatively. The percent overshoot was defined as

18 $${n}_{os}=\underset{t}{\mathrm{max}}({y}_{norm}(t)-1)\times 100,{ n}_{os}\ge 0$$

representing the relative amount by which the normalized response exceeds its final value. The steady-state error was calculated as

19 $${n}_{se}=\mid {y}_{norm}({t}_{sim})-1\mid \times 100,{t}_{sim}=20\hspace{0.17em}min$$

to measure the deviation from the target level at the end of the simulation. The normalized settling time was determined from

20 $$n_{st} = \min \left\{ {t:|y_{norm} (\tau ) - 1| \le 0.02\forall \tau \in [t,t_{sim} ]} \right\}$$

as the earliest instant when the response remains within the $\pm 2\%$ band. Finally, the rise time was obtained by

21 $$n_{rt} = t_{90} - t_{10} ,t_{p} = \min \left\{ {t:y_{norm} (t) \ge p} \right\},p \in \{ 0.1,0.9\}$$

expressing the time required for the normalized output to increase from 10 to 90% of its final value.

### Objective function

To merge the time-domain performance indicators into a single evaluation score, a modified version of the Zwe Lee Gaing’s (ZLG) function was employed as the objective function^31^:

22 $$OF=\frac{\left(1-{e}^{-\varphi }\right)}{100}\left({n}_{os}+{n}_{se}\right)+{e}^{-\varphi }({n}_{st}-{n}_{rt})$$

**Fig. 4:**
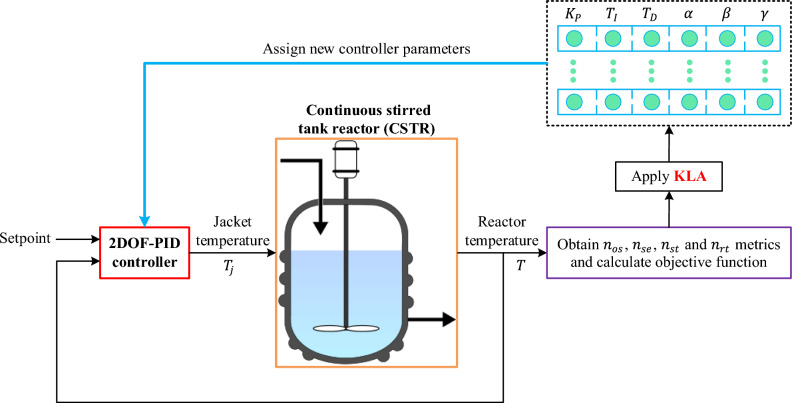
Block diagram of the KLA-optimized 2DOF-PID control scheme for the CSTR system.

The first term penalizes excessive ${n}_{os}$ and ${n}_{se}$ in percentage form, while the second term rewards fast yet stable transitions by producing smaller values when both ${n}_{st}$ and ${n}_{rt}$ are low. The difference establishes a balance between response speed and smoothness within the same normalized band. The weighting between these components is governed by the balance coefficient $\varphi$; larger values reduce the influence of speed, whereas smaller $\varphi$ values make the speed component more dominant. In this study, the equilibrium factor was set to $\varphi =1$ following the original formulation of the ZLG performance index in the literature, which provides a balanced weighting between the performance terms. The optimization goal is to minimize the objective function, $OF$. The complete optimization loop, illustrated in Fig. 4, proceeds through the following stages. The KLA samples an initial population of candidate parameter vectors $\theta =\left[{K}_{P},{T}_{I},{T}_{D},\alpha ,\beta ,\gamma \right].$

Each candidate is applied to the CSTR model, where a step input of 20 K is introduced at $t=1\hspace{0.17em}min$. The normalized response ${y}_{norm}(t)$ is evaluated to compute the four performance indices ${n}_{os}$, ${n}_{se}$, ${n}_{st}$, and ${n}_{rt}$. Invalid simulations or constraint violations incur a significant penalty added to $OF$. The resulting objective values are fed back to the KLA, which then generates updated candidate parameters. When the convergence criterion is satisfied, the best parameter set $\widehat{\theta }$ is selected and stored for experimental validation.

## Simulation results and discussion

The effectiveness of the proposed control method was evaluated in terms of transient performance and stability through simulation studies. In this case, the designed 2DOF-PID controller was tested with different sets of numbers obtained using various methods to improve performance. The evaluation process considered the ${n}_{rt}$, ${n}_{st}$, ${n}_{os}$, and ${n}_{se}$ performance indices as key metrics. The data procured in the simulation environment were scrutinised through both time-domain responses and statistical consistency analyses. The findings of the optimisation process are presented below, along with a quantitative assessment of the achieved control performance.

### Statistical consistency of optimization results

The effectiveness of the proposed approach was evaluated through a comparative optimisation study using several well-established techniques. Each method was tested under identical experimental conditions. This procedure was implemented to ensure a fair comparison among all optimization algorithms. The methods tested were KLA, AOO, PO, COA, and DMO. All algorithms were executed in 25 independent runs with the same population size (${N}_{pop}=30$) and the same maximum number of iterations ($ite{r}_{max}=100$).

**Fig. 5:**
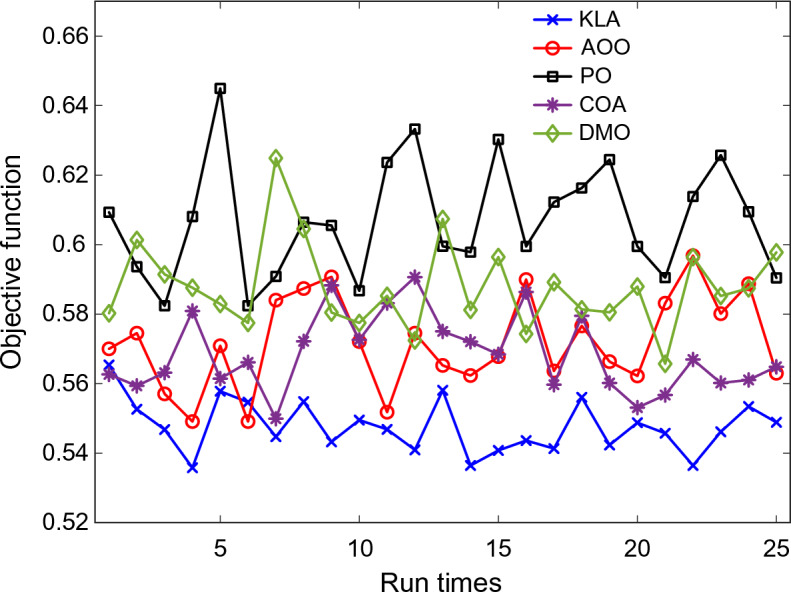
Comparison of objective function values for different optimization algorithms over 25 independent runs.

As demonstrated in Fig. 5, the distribution of objective function values derived from these runs is presented. The findings clearly show that the KLA achieved more modest, consistent objective values than other methods. This behaviour indicates that the algorithm is relatively insensitive to initial conditions and consistently produces similar results, despite being random.

**Fig. 6:**
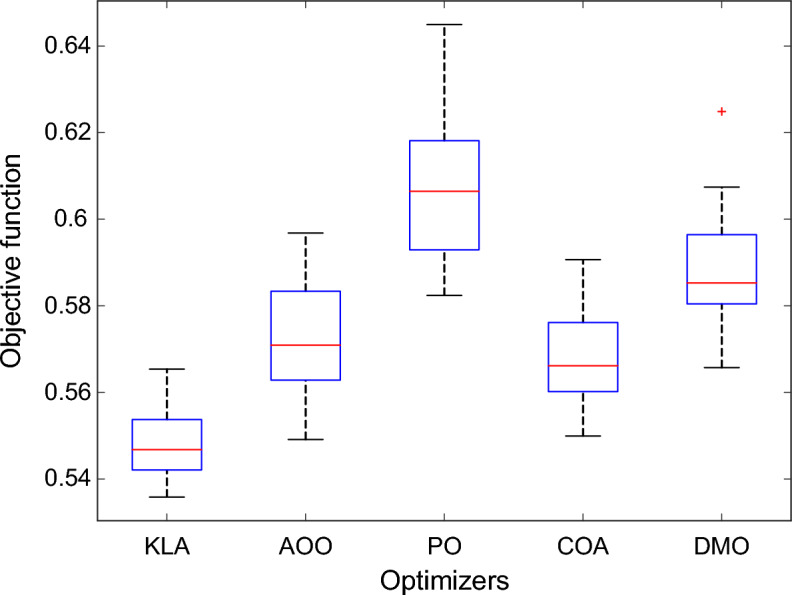
Statistical distribution of objective function values for different optimization algorithms.

The results show that the PO and DMO algorithms have wider changes in their objective function values. This indicates a higher level of randomness and instability during the search process. The boxplot in Fig. 6 clearly illustrates this behaviour. The allocation linked to KLA is concentrated within a limited scope, whereas the PO outcomes are more widely dispersed and have a higher median value. Table 2 outlines the numerical specifics of these statistical disparities.

**Table 2:** Statistical comparison of optimization performance metrics for different algorithms.

Statistical metric	KLA	AOO	PO	COA	DMO
Minimum	**0.5358**	0.5491	0.5824	0.5499	0.5657
Maximum	**0.5654**	0.5968	0.6449	0.5906	0.6249
Median	**0.5468**	0.5709	0.6065	0.5661	0.5853
Average	**0.5476**	0.5719	0.6071	0.5686	0.5879
Standard deviation	**0.0075**	0.0134	0.0166	0.0112	0.0127
Rank	**1**	3	5	2	4

All tested algorithms achieved an average objective function value of at least 0.5476, with a standard deviation of only 0.0075. The KLA had the lowest mean value among all the algorithms evaluated. Conversely, the PO algorithm yielded an average of 0.6071, accompanied by a conspicuously elevated standard deviation of 0.0166. When selecting an evaluation tool, it is important to consider that the KLA produces more consistent and reliable results when tested repeatedly. The foremost position was attained by KLA, with COA, AOO, DMO and PO following in that order.

The Wilcoxon rank-sum test^32^ results, presented in Table 3, confirm that these differences are statistically significant. All p-values below 0.05 indicate that KLA performs significantly better than the other algorithms.

**Table 3:** Wilcoxon rank-sum test results comparing KLA with other optimization algorithms.

Nonparametric metric	KLA versus AOO	KLA versus PO	KLA versus COA	KLA versus DMO
p-value	1.7735E − 05	1.2290E − 05	1.3898E − 05	1.2290E − 05
Winner	KLA	KLA	KLA	KLA

### Convergence analysis

**Fig. 7:**
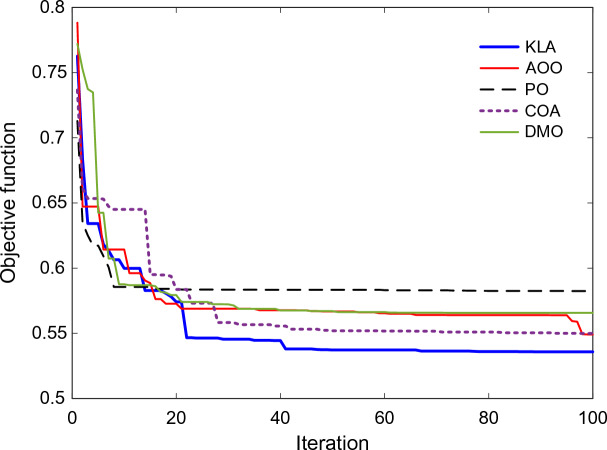
Evolution of objective function values over iterations for different optimization algorithms.

As shown in the convergence profiles in Fig. 7, the KLA consistently reduced the objective function with each successive iteration. A marked improvement was obvious during the first 20 repetitions, after which the curve slowly steadied and reached a steady value around the 60th repetition. In contrast, the residual algorithms exhibited a slower rate of convergence. In fact, a number of them, most notably PO and DMO, hit a dead end at high target levels before the set timeframe had come to an end.

The KLA trend shows a slow, steady drop, with a mix of looking at the world and improving things locally. This behaviour increases the likelihood of finding the global optimum while decreasing the likelihood of finding local minima. As a result, the KLA mechanism provides a useful technique for calculating exact controller gains.

### Optimized controller parameters

Table 4 lists the optimal 2DOF-PID parameters obtained from each optimization algorithm. The set derived through the KLA differs noticeably from those of the other methods. In this configuration, the derivative time constant is relatively large (${T}_{D}=0.90335$ min), whereas the integral time constant is relatively small (${T}_{I}=0.0563$ min). This combination results in a rapid temperature increase and quick stabilization without further delay. The proportional gain ${K}_{P}=0.44037$ is still safely bounded, preventing actuator saturation.

**Table 4:** Optimized 2DOF-PID controller parameters obtained by different optimization algorithms.

Controller parameter	Range	KLA	AOO	PO	COA	DMO
${K}_{P}$	$[0.01, 0.5]$	0.44037	0.49986	0.43850	0.46865	0.45838
${T}_{I}$	$[0.02, 0.4]$	0.056272	0.39441	0.37891	0.36432	0.39709
${T}_{D}$	$[0.01, 1.5]$	0.90335	0.065406	0.044473	0.035987	0.032140
$\alpha$	$[0.05, 0.2]$	0.15802	0.15354	0.19571	0.094342	0.19427
$\beta$	$[0.5, 2]$	1.6572	1.8185	1.3760	1.3189	1.7251
$\gamma$	$[0.1, 10]$	2.1235	6.8455	6.6208	7.7975	5.7133

The tracking parameters were estimated as $\beta =1.6572$ and $\gamma =2.1235$. These parameters provide an assertive controller response to changes in the reference while limiting overshoot in the process. With this tuning, KLA fully exploits the two degrees of freedom in the controller structure to enhance both tracking and regulation performance. The normalized performance indices in Table 5 also confirm the quantitative effect of these parameters on the entire system.

**Table 5:** Normalized stability performance metrics obtained by different optimization algorithms.

Normalized stability metric	KLA	AOO	PO	COA	DMO
Rise time ${n}_{rt}$ (min)	0.1692	1.2566	1.7375	1.5367	1.4179
Settling time ${n}_{st}$ (min)	1.6179	2.7151	3.2981	2.9972	2.9300
Overshoot ${n}_{os}$ (%)	0.4550	1.9934	1.3156	1.9910	1.4879
Steady-state error ${n}_{se}$ (%)	2.0217E − 07	2.2560E − 07	2.1947E − 07	2.8541E − 07	2.3261E − 07

### Dynamic response of the CSTR system

**Fig. 8:**
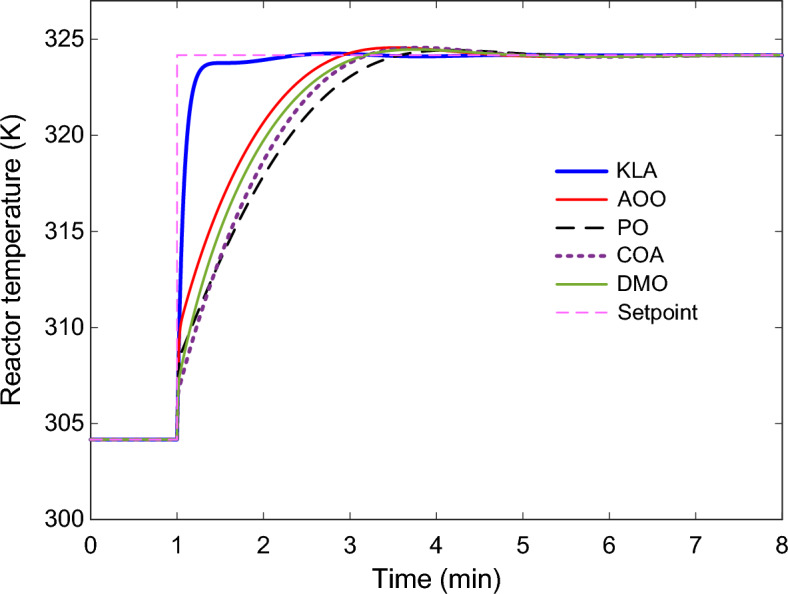
Reactor temperature responses obtained using 2DOF-PID controllers optimized by different algorithms.

**Fig. 9:**
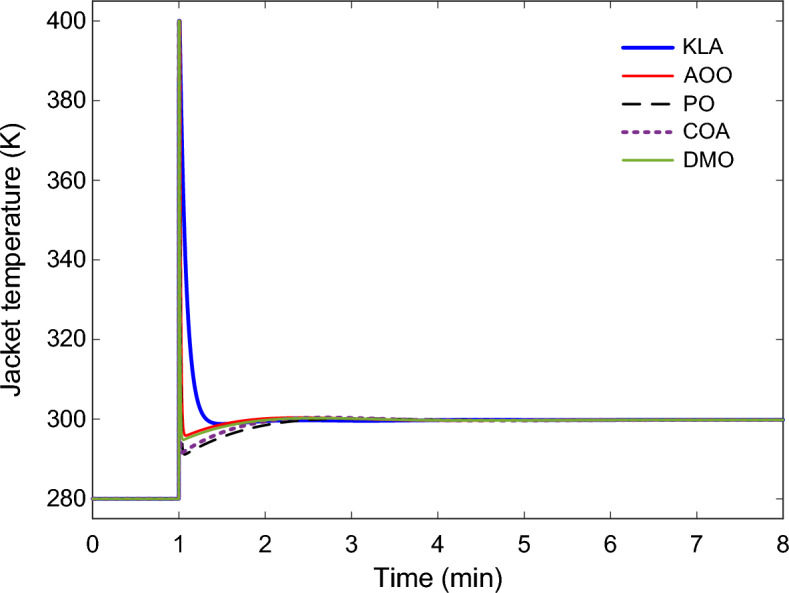
Jacket temperature profiles for the CSTR under 2DOF-PID controllers optimized by different algorithms.

**Fig. 10:**
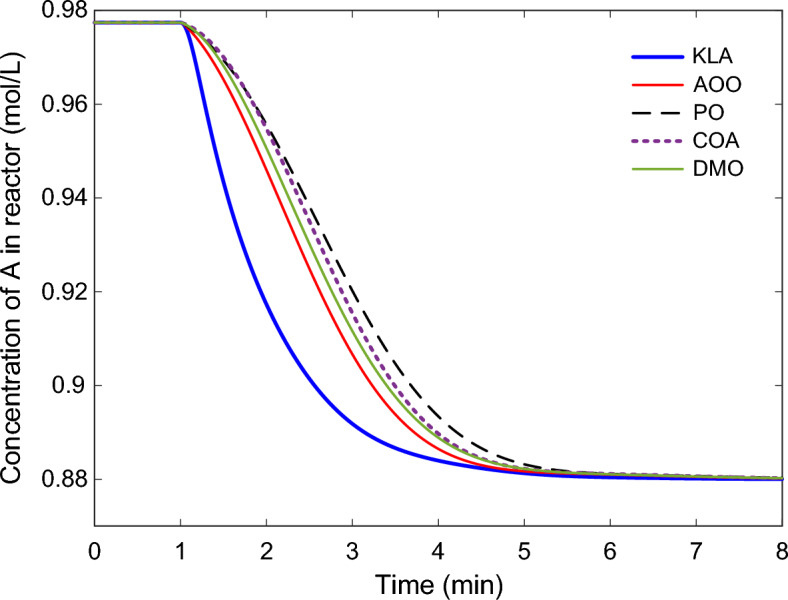
Time evolution of reactant A concentration in the CSTR under different optimized controllers.

The dynamic responses of the 2DOF-PID controller optimized with KLA in the CSTR system are presented in Fig. 8, Fig. 9, and Fig. 10. The reactor temperature response shown in Fig. 8 shows the behavior of all algorithms when the setpoint is increased by 20 K from 304.17 K to 324.17 K. The KLA-based controller reached steady state in the shortest time and significantly reduced the rise time. The system reached steady state in approximately 1.6 min, with an ${n}_{os}$ below 0.5%. This time was extended to 2.7–3.3 min for the other algorithms. This observation is consistent with the numerical stability metrics in Table 5.

The combination of the low ${T}_{I}$ and high ${T}_{D}$ values obtained by the KLA enabled faster heat transfer during the transient phase. As shown in Fig. 9, the variation of the jacket temperature ${T}_{j}$ indicates that the KLA produced a shorter yet higher-amplitude heating pulse compared to the other algorithms. This brief energy burst enabled the reactor to reach the target temperature rapidly, after which the system smoothly transitioned to a stable regime. Consequently, the control signal applied to the heater exhibited neither excessive oscillations nor saturation behavior. In addition, the transient, high-amplitude pulse observed in the jacket temperature profile should be interpreted as a short-duration energy injection required to overcome the reactor’s thermal inertia. Considering the overall heat-transfer coefficient and reactor volume given in Table 1, the corresponding heat duty remains within the practical operating range of industrial electric or steam-based jacket heating systems. No sustained actuator saturation or physically unrealistic temperature excursion is observed. Therefore, the control action remains feasible for real industrial heater implementations.

The concentration response of component $A$ in the reactor, ${C}_{A}\left(t\right),$ is presented in Fig. 10. The KLA controller reached the equilibrium concentration more quickly due to the rapid increase in reaction temperature. Systems controlled with the AOO, COA, and DMO algorithms reached this conversion level later, while the PO control produced the slowest response. This demonstrates that the KLA-optimized controller improved both thermal and reaction stability.

### Numerical stability analysis

Table 5 presents a numerical comparison of the stability metrics obtained from the reactor temperature response in Fig. 8. The ${n}_{rt}$, ${n}_{st}$, ${n}_{os}$ and ${n}_{se}$ values clearly demonstrate that KLA demonstrates superior performance in all metrics.

KLA’s normalized ${n}_{rt}$ is the lowest at 0.1692 min, indicating that the reactor reaches the target temperature approximately 7–10 times faster than other methods. The ${n}_{st}$ is found to be 1.6179 min, achieving stability approximately 45% faster than its closest competitor, COA. The ${n}_{os}$ is 0.455%, indicating that the system response is balanced without excessive energy accumulation. The ${n}_{se}$ is at a negligible level of 2.02 × 10^–7^% and is practically equivalent to zero.

Despite these values, the PO algorithm exhibited the poorest performance with a ${n}_{rt}$ of 1.7375 min and a ${n}_{st}$ of 3.2981 min. The AOO, COA, and DMO algorithms also exhibited longer transient durations compared to KLA. Therefore, when all metrics in Table 5 are evaluated together, it is confirmed that KLA provides the most effective control of the system, with respect to both speed and stability.

A quantitative comparison makes the advantage of KLA even clearer beyond its parameter-free nature. KLA achieves around 46% less settling time and almost 89% better rise time than its nearest rival. For the objective function, the KLA mean value is approximately 3.7% better than COA and 9.8% better than PO. These results indicate that KLA’s superiority lies not only in its simple structure but also in tangible performance enhancements.

### Variable reference tracking performance

**Fig. 11:**
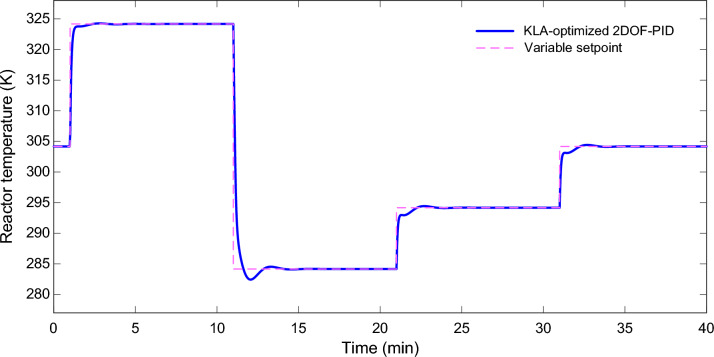
Reactor temperature response under variable setpoint tracking using the KLA-optimized 2DOF-PID controller.

Fig. 11 shows the performance of the 2DOF-PID controller optimized by KLA for a time-varying reference temperature trajectory. The reactor temperature tracked ramping set values with minimal delay and no overshoot. This means the controller has stable, robust performance not only at a single operating point but across the entire operating region. There are no oscillations and no integral windup in the responses. At each step, the controller pushed the system to a new equilibrium point really quickly, so it did not require excessive energy. The simulation results demonstrate that the KLA-optimized 2DOF-PID architecture is very effective in non-linear chemical reactors.

### Disturbance rejection against feed temperature fluctuations

To more accurately assess the disturbance rejection performance of the proposed control scheme, ${T}_{f}$ was perturbed externally. In realistic chemical reactor operation, the feed temperature varies due to changes in the upstream process, disturbances in the heat exchangers, or environmental conditions. Such perturbations can strongly influence reactor temperature because reaction kinetics are tightly coupled with thermal dynamics in CSTR systems.

**Fig. 12:**
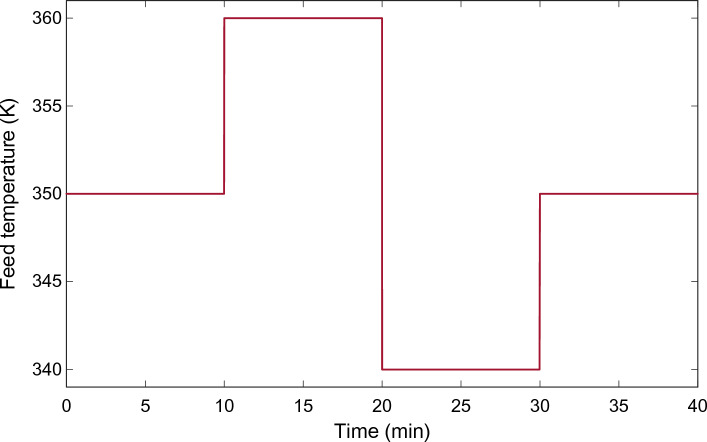
Step disturbance profile applied to the feed temperature ${\mathrm{T}}_{\mathrm{f}}$ to evaluate disturbance rejection performance.

Fig. 12 shows the feed temperature profile used to impose realistic disturbance conditions. The nominal feed temperature was at first held constant at 350 K. At $t=10$ min, the feed temperature was increased to 360 K to introduce a positive disturbance. This perturbation lasted until $t=20$ min, when a negative perturbation was imposed by lowering the feed temperature to 340 K. Again, at $t=30$ min, the feed temperature was set back to its nominal value of 350 K. The above sequence of disturbances was intended to simulate heating and cooling perturbations as could be experienced in actual industrial operations.

**Fig. 13:**
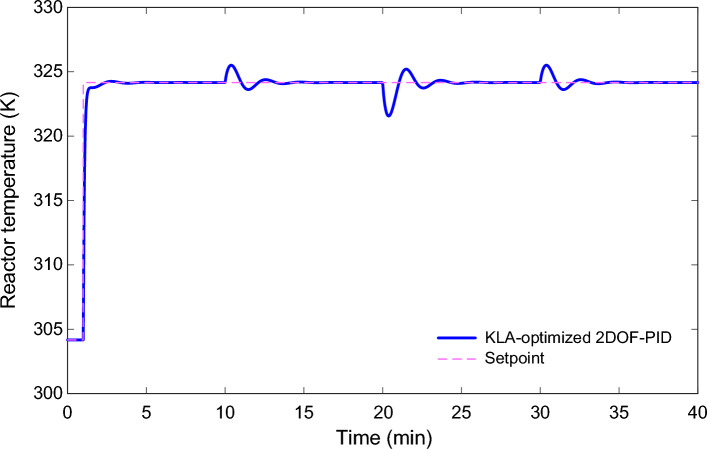
Reactor temperature response under feed temperature disturbances using the KLA-optimized 2DOF-PID controller.

Fig. 13 shows the corresponding reactor temperature response using the KLA-based 2DOF-PID controller. As shown, the controller successfully attenuates the effect of feed temperature perturbations on the reactor temperature. The reactor temperature at a positive disturbance at $t=10$ min only experiences a small transient deviation from the setpoint and then quickly returns to the desired operating value. A similar situation is observed during the negative perturbation at $t=20$ min, where the system briefly deviates before quickly stabilizing. The transient deviations in are not large in magnitude and duration, which implies that the proposed controller also possesses strong disturbance attenuation capability. In particular, the oscillations after disturbance events are small and rapidly damped, indicating good regulation performance despite the non-linear thermal reactor dynamics.

In general, the KLA-optimized 2DOF-PID provides stable and accurate temperature control despite large variations in the feed temperature. This behavior demonstrates that the suggested optimization-based tuning method is robust and can be applied in real industrial CSTR applications, even in the presence of unforeseen external disturbances.

### Robustness under parametric uncertainty

In the normal course of chemical reactor operation, process parameters deviate from their nominal values due to model errors, equipment aging, heat-transfer fouling, and changes in reaction kinetics. These uncertainties have a considerable impact on the dynamics of non-linear CSTR systems. Hence, in addition to the disturbance rejection performance analysis, it is also necessary to consider the robustness of the designed controller under parametric uncertainty.

To study this aspect, several critical process parameters were perturbed from their nominal values. The considered uncertainty scenarios are summarized in Table 6. Three representative cases were considered. In Case (a), the overall heat-transfer coefficient–area product $UA$ was increased by 20%. In Case (b), the inlet flow rate $F$ was decreased by 10%. In Case (c), the activation energy over gas constanst term $E/R$ was increased by 5%. These changes were selected because they reflect realistic deviations often encountered during industrial reactor operation.

**Table 6:** Parametric uncertainty scenarios applied to the CSTR model and the resulting steady-state operating points.

Case	System parameter	Change	Steady-state operation points of the system
(a)	$UA$	+ %20	$T=300.9350$ K and ${C}_{A}=0.9833$ mol/L
(b)	$F$	− %10	$T=302.3921$ K and ${C}_{A}=0.9788$ mol/L
(c)	$E/R$	+ 5%	$T=302.9664$ K and ${C}_{A}=0.9952$ mol/L

**Fig. 14:**
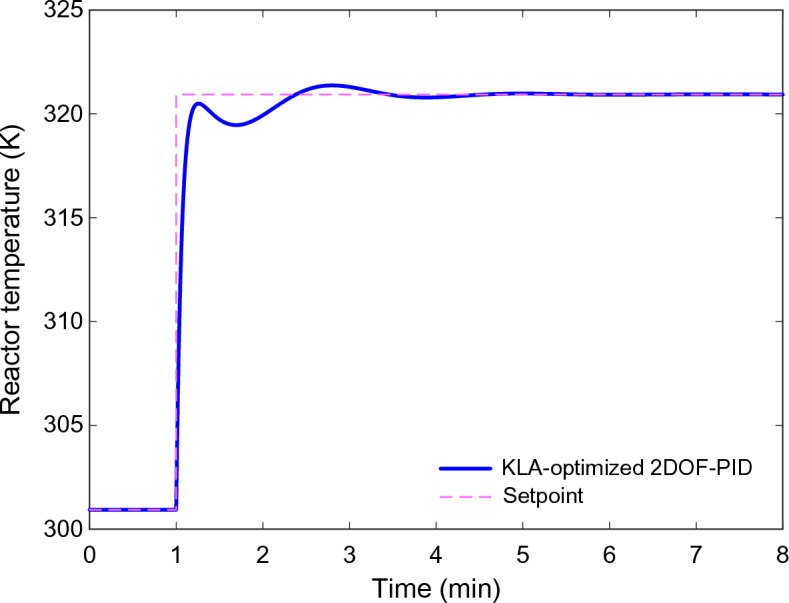
Reactor temperature response under parametric uncertainty in the heat-transfer coefficient–area product ($\mathrm{UA}$, + 20%).

The steady-state operating points corresponding to each parameter variation are listed in Table 6. As shown, there is a slight shift in the system equilibrium after changing the parameters, affecting both the reactor temperature and the reactant concentration. These modifications introduce additional non-linear effects that may challenge controller performance. The dynamic temperature responses of the CSTR under the three uncertainty scenarios are illustrated in Fig. 14–Fig. 16. Although parameter deviations are present, the KLA-optimized 2DOF-PID controller maintains stable tracking performance and successfully drives the reactor temperature to the desired setpoint. In all three cases, the reactor temperature approaches the reference value smoothly without instability or sustained oscillatory behavior.

**Fig. 15:**
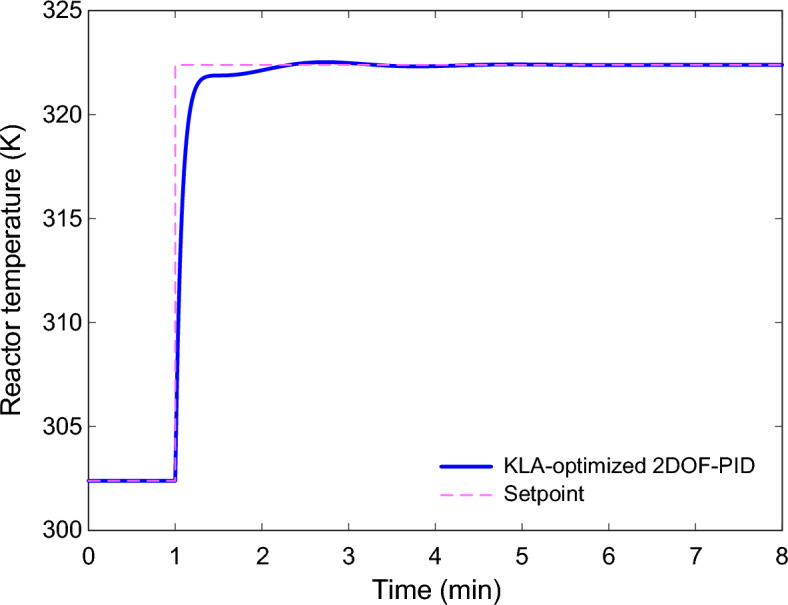
Reactor temperature response under parametric uncertainty in the inlet flow rate ($\mathrm{F}$, − 10%).

**Fig. 16:**
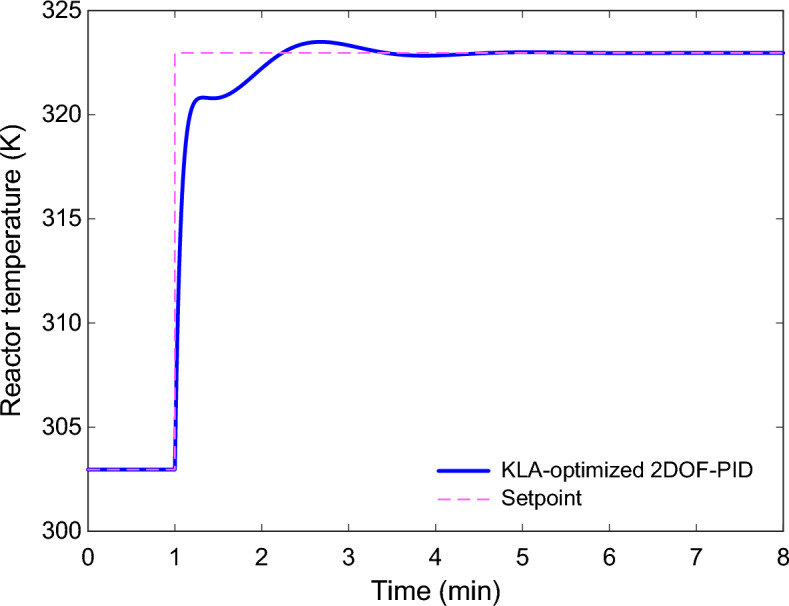
Reactor temperature response under parametric uncertainty in the activation energy over gas constant term ($\mathrm{E}/\mathrm{R}$, + 5%).

In Case (a), the system is slightly slower in the transient due to modified thermal dynamics, since the heat-transfer ability is enhanced. However, the controller compensates for this variation and stabilizes the regulation with a small overshoot, as shown in Fig. 14. In Case (b), with a decreased inlet flow rate, the reactor dynamics become less aggressive. The controller becomes more damped, resulting in a smoother response, as shown in Fig. 15. In Case (c), the reactor’s thermal sensitivity changes because an increase in $E/R$ alters the reaction kinetics. Even in this situation, the proposed controller ensures stable system performance and rapidly returns the system to the target temperature, as shown in Fig. 16.

A quantitative comparison of the corresponding performance indices is listed in Table 7. The rise time, settling time, overshoot, and steady-state tracking error values indicate that acceptable control performance is maintained across all uncertainty cases. While small differences in the transient behavior are observed, the steady-state error is negligible in each case, indicating that the reactor temperature is correctly regulated.

**Table 7:** Performance metrics obtained under parametric uncertainty conditions.

Normalized stability metric	Case (a)	Case (b)	Case (c)
Rise time ${n}_{rt}$ (min)	0.1264	0.1707	0.6039
Settling time ${n}_{st}$ (min)	2.9348	1.8264	2.9515
Overshoot ${n}_{os}$ (%)	2.1751	0.6578	2.6478
Steady-state error ${n}_{se}$ (%)	1.1498E − 08	3.6499E − 08	2.5821E − 09

Based on these results, the 2DOF-PID controller optimized by the proposed KLA method can be considered robust to typical parameter uncertainties in CSTR processes. The controller remains stable and provides good transient responses under large changes in the heat-transfer properties, flow rate, and reaction kinetics. These results confirm the feasibility of the proposed scheme for use in real industrial environments where exact parameter values are not always known.

## Conclusion

The study proposes a 2DOF-PID controller optimized using the KLA for temperature regulation in a CSTR characterised by non-linear dynamics and strong heat-transfer coupling. The controller configuration has been developed based on a mathematical model of the reactor that incorporates both mass and energy balances. The KLA uses the ideas of KCL, which are often used to analyse electrical circuits, to develop a search process focused on balancing energy. This strategy offers an operation that is entirely unconstrained by parameters, stable performance, and rapid convergence by modelling the interactions among candidate solutions as current-flow behaviour within the search space.

In comparative evaluations, the KLA achieved lower objective-function values than the AOO, PO, COA, and DMO algorithms, and outperformed them in stability metrics such as ${n}_{rt}$, ${n}_{st}$, ${n}_{os}$ and ${n}_{se}$. The temperature response of the KLA-optimized controller reached steady-state within 1.6 min, maintaining an ${n}_{os}$ below 0.5%. These results highlight KLA’s strong global search capability for non-linear energy-balance optimization problems. The improvements achieved by KLA are quantitatively significant, particularly in settling time and objective function reduction, confirming that its advantage extends beyond being merely parameter-free.

The KLA requires no settings, so the user does not need to define control variables, which makes it useful in practice. High accuracy is delivered with low computational demand by its population-based structure. At the same time, stability in the optimization process is ensured, and the current–voltage analogy suppresses fluctuations in the objective function. The results show that KLA can be used not only to improve things but also to adjust control parameters based on ideas about how the world works. These results demonstrate that the KLA-based 2DOF-PID design provides reliable, overshoot-free, and highly stable control performance in non-linear thermal processes.

Future work plans to apply KLA to more complex multivariable processes by combining it with adaptive or hybrid structures. Furthermore, testing the algorithm in real-time hardware-based systems is an important research direction to demonstrate the method’s practical applicability.

## Data Availability

All data generated or analysed during this study are included in this published article.
